# Optimizing instilled drug delivery: a scoping review of microdrops in ophthalmology

**DOI:** 10.1007/s00417-025-06773-1

**Published:** 2025-02-26

**Authors:** Aikaterini K. Seliniotaki, Tatiana Tziola, Maria Lithoxopoulou, Argyrios Tzamalis, Nikolaos Ziakas, Asimina Mataftsi

**Affiliations:** 1 2nd Department of Ophthalmology, School of Medicine, Faculty of Health Sciences, Aristotle University of Thessaloniki, Papageorgiou General Hospital, N.Efkarpia, Thessaloniki, 56429 Greece; 2 2nd Department of Neonatology, School of Medicine, Faculty of Health Sciences, Aristotle University of Thessaloniki, Thessaloniki, Greece

**Keywords:** Eyedrops, Micro-dose, Minidrops, Mydriatics, Ocular hypotensive drugs, Pharmacokinetics, Adults, Children, Infants, Newborns

## Abstract

Eyedrop instillation constitutes the most commonly used ocular drug delivery method that serves for both diagnostic and therapeutic purposes. Ocular disposition and bioavailability of instilled drugs depend on the anatomy and physiology of the ocular surface as well as the physicochemical properties of the active agent. Intraocular bioavailability is positively associated with the amount of drug available onto the ocular surface and the precorneal residence time. Concerns are raised regarding systemic absorption of the instilled drugs intraocularly, percutaneously, via the conjunctiva, through the nasolacrimal system, or through the nasal, oral, and gastrointestinal mucosa. Special considerations exist regarding the anatomical features and the limited pharmacokinetic data on the pediatric population that complicate further the efficacy and systemic toxicity of the instilled medications. Both preclinical and clinical studies propose the reduction of the instilled drop volume, in the form of microdrops, as a means to enhance intraocular bioavailability of topically applied drugs, while minimizing patient discomfort and systemic adverse events. We summarize existing data on the clinical application of microdrops in a wide age range, from preterm infants to elderly adults. Studies regarding microdrops of mydriatics and ocular hypotensives show promising results in optimizing the provided everyday care.

## Abstract (highlights)

**Key messages:**

*****What is known***:**

- Eyedrops are part of the everyday care and are not deprived of adverse events, so looking for the optimized instilled drug delivery is of utmost importance.

*****What is new***:**

- Microdrops of mydriatics and ocular hypotensive drugs have been used efficiently in infants, children, and adults for diagnostic and therapeutic purposes, while reducing systemic toxicity and excipient-related adverse events.

## Introduction

Eyedrop instillation has been part of the ophthalmic care even since the times of Ancient Egypt [1], and is the most commonly used ocular drug delivery method until nowadays, accounting for approximately 90% of the marketed ophthalmic drug formulations [2, 3]. As any other form of topical treatment, for instance in dermatology or otorhinolaryngology, eyedrops constitute a less intrusive route of drug delivery that serves for both diagnostic and therapeutic purposes, and is characterized by their ease of administration and low cost [2, 3]. 

Topically instilled drugs are intended to act on the ocular surface or to exert an effect on some internal ocular structure after corneal penetration. The limited ocular bioavailability, the risk of toxicity, and the poor patient compliance, are major disadvantages of this type of drug delivery [4–6]. Anatomical and physicochemical barriers of the eye, the restrictive absorption area, low corneal permeability, metabolism or binding of drug molecules with proteins in the tear fluid, and drainage into the nasolacrimal system, raise unique challenges for scientists to achieve the optimal drug concentration on the targeted ocular tissues while minimizing drug-induced adverse events [3, 7–9]. 

Reducing the instilled drop volume has been proposed as a method of enhancing corneal penetration and improving intraocular bioavailability of topically applied drugs, while minimizing patient discomfort and systemic adverse events [10–12]. The use of “microdrops”, or else called “nano-drops”, “mini-drops”, or “micro-doses”, has been investigated for various ocular medications including mydriatics, cycloplegics, beta-blockers, and ocular hypotensive drugs [11, 13–16]. Experiments in animal models and clinical studies yielded promising results in a wide age range, from preterm infants to elderly adults [10, 11, 13–17]. 

We compile data on the anatomy and physiology of the ocular surface, the pharmacokinetics of ocular drugs, the rationale for using microdrops, and their clinical applications. Both preclinical and clinical studies were considered, trying to present the translation from bench to bedside.

## Methods

### Literature search

Literature search strategies were developed using text words related to microdrop instillation. PubMed was searched up to 29th December 2024, without a starting point limitation. No restrictions were applied. The electronic database search was supplemented by searching in ClinicalTrials.gov for ongoing studies, as well as through scanning the reference lists of included studies for additional eligible studies. The search string on PubMed is presented in Appendix.

#### Inclusion criteria

studies evaluating the use of reduced eyedrop volume, either in animal models or in humans, with or without the presence of a control group, i.e., a group receiving standard drops.

#### Exclusion criteria

other forms of ocular drug delivery except for instilled eyedrops (solutions or suspensions), e.g., gels and ointments.

## Anatomy and physiology of the ocular surface

In 1662, Danish scientist Niels Stensen was the first to describe that tears are secreted by the lacrimal glands [18], while the term “Lacrimal Functional Unit” was first introduced in 1998 for describing the relationship between the lacrimal gland, the ocular surface, and their interconnecting innervation [19]. 

### Lacrimal secretion and drainage

The lacrimal apparatus is composed of a secretory part, also known as the glandular section, and a drainage part, the nasolacrimal system. Tear fluid is primarily supplied by the secretions of the main lacrimal gland, the accessory lacrimal glands of Wolfring and Krause, the Meibomian tarsal glands, conjunctival goblet and epithelial cells, corneal epithelial cells, and the cells of the nasolacrimal duct epithelium [20, 21]. Tear fluid composition is subjected to homeostatic processes, thus presenting variations under different situations including open- and closed-eye phases (prolonged eye closure) of the diurnal cycle, basal and reflex tear secretion, wound healing subsequent to trauma or refractive surgeries, as well as in pathologic conditions, including dry eye disease, infectious, inflammatory, or systemic diseases [22–25]. In consequence, advances in proteomics, metabolomics, and lipidomics technologies are oriented towards utilizing tear fluid as a disease biomarker and ultimately serving personalized medicine [24]. 

The *main and accessory lacrimal glands* secrete electrolytes, water, and a variety of proteins, peptides, and glycopeptides, that formulate the aqueous component of the preocular tear film [20]. Interestingly, proteomics research on human tear fluid established the existence of more than 1500 proteins in this extremely complex biological mixture [24, 26, 27]. The superficial lipid layer is produced by the *Meibomian glands* and is composed of wax monoesters and sterol esters, several types of diesters, hydrocarbons, triglycerides, diglycerides, free sterols, including cholesterol, free fatty acids, and polar lipids, including phospholipids [25, 28–30]. The Meibomian gland fluid presents significant between-person variability, depending on many neuronal, hormonal, and vascular factors [20, 29, 31]. Changes in the level of sex hormones, mainly androgens, and the activity of lipases produced by bacteria, may alter the lipid profile resulting in tear film destabilization and increased tear evaporation [25, 29, 31]. 

The inner mucus layer is the product of several different types of cells. The *conjunctival and corneal epithelial cells* produce membrane bound mucins, i.e., MUC1, MUC4, MUC16, and antimicrobial peptides to react against pathogens [20]. The *conjunctival goblet cells* secrete the main component of tear mucus, i.e. MUC5AC, as well as MUC2 and trefoil family factor peptides 1 and 3 (TFF1 and TFF3). The TFF peptides influence the rheological properties of the tear film, present antiapoptotic properties, promote corneal epithelial cell migration, and induce cell scattering [20, 32, 33]. Finally, the *acinar and excretory duct cells of the lacrimal gland* produce membrane bound and secretory mucins, i.e., MUC1, MUC4, MUC5AC, MUC5B, MUC7. The latter two have been shown to bind bacteria, contributing to the immunological properties of the tear film [20, 30]. 

The lacrimal gland secretion is highly regulated, controlling the tear volume and composition [21, 34, 35]. Subconscious stimulation of the sensory nerve endings in the corneal and conjunctival epithelia, in response to ocular surface demands and environmental changes, such as temperature, humidity, mechanical forces, and irritant chemicals or pathogens, generates afferent nerve impulses that reach the lacrimal nuclei in the mid-brain. The mid-brain also processes input (e.g., emotional) from other centers to produce a graded output [34, 35]. Efferent innervation towards the main and accessory lacrimal glands and excretory ducts is served by both the parasympathetic and sympathetic nervous system to regulate basal and reflex tear secretion. Parasympathetic innervation predominates, both anatomically and functionally, promoting tear secretion, while sympathetic innervation only supports basal secretion [21, 34, 35]. 

The nasolacrimal system is not purely a drainage tube and is subjected to a dynamic autoregulation. *Nasolacrimal duct epithelial cells* are active in absorbing tear fluid components, and in secreting MUC1, MUC2, MUC4, MUC5AC, MUC5B, MUC7, TFF1, TFF3, lysozyme, lactoferrin, and phospholipase A2. Thus, they facilitate tear outflow and contribute to antimicrobial defense [20, 21]. The nasolacrimal duct lumen diameter is directly dependent on the state of a *densely innervated cavernous body*, rich in specialized arteries and veins, which surrounds the lacrimal sac and the nasolacrimal duct [36–38]. Vasoconstriction of the cavernous body blood vessels, induced by sympathetic stimulation, dilates the lumen of the lacrimal passage. On the contrary, swelling of the cavernous body through the parasympathetic stimulation interrupts tear drainage, inducing epiphora, which serves as a protective mechanism against foreign bodies and is also related to emotional responses [36–38]. 

As expected, *physical factors* including gravity, respiration, and evaporation are involved in the drainage of tears via the lacrimal puncta to the upper and lower lacrimal canaliculi, the lacrimal sac, and the nasolacrimal duct [20]. Finally, contraction of the lacrimal portion of the orbicularis muscle with *blinking* distends the lacrimal sac and generates a pumping action of the canaliculi, assisting the capillary attraction of tears. It is estimated that 2 µl of tears are pumped out at each blink [20, 37–40]. 

### Tear film, turnover rate, and break-up time

The total tear volume is divided into the following continuous compartments: the preocular tear film, the upper and lower tear menisci, and the upper and lower cul-de-sac/fornices [41, 42]. The tear film serves to coat, lubricate, nourish, and immune protect the ocular surface, and maintain its homeostasis, thus preserving consistent clarity of vision [43]. The menisci serve as a reservoir of tear fluid that supplies and re-forms the tear film at each blink or stores the accumulated reflex tears in case of lacrimal drainage obstruction or eyedrop instillation [44, 45]. 

Six decades ago, Niels Ehlers provided an estimate of the *size of the human conjunctival sac*. The depth of the inferior and superior fornix, corresponding to the middle of the free eyelid margin, was found to be 9–11 mm and 14–16 mm, respectively. He also measured the level of conjunctival distension with eyelids closed and calculated the total area of the conjunctival sac per eye to be 16 cm^2^ [46]. A year later, Mishima et al. ascertained the normal *tear volume* at an average of 6.2 ± 2 µl, as measured in 16 men and 21 women between 20 and 89 years of age [41]. The average normal tear flow rate was calculated to be 1.2 µl per minute, with a range of 0.5 µl to 2.2 µl per minute [41]. The normal tear turnover rate was estimated at 16% per minute, while it increased largely after an eyedrop instillation, to almost 80% per minute, presumably as a reaction for preventing tear overflow [41]. The maximum quantity of tears that could be contained in the cul-de-sac, in the upright position, without overflow, was about 30 µl, but if blinking was permitted, then the eye could only hold approximately 10 µl [41]. The *tear meniscus height* also serves as an indicator of total tear volume, and an average measurement was found to be 0.19 mm in the primary, and 0.25 mm in the up-gaze position [42, 47]. 

*Tear film stability* is primarily associated with the amount of the aqueous tear layer depletion rate due to evaporation, which in turn depends on the quality of the tear lipid layer [45, 48]. However, there are multiple parameters that also affect tear film stability, including age, gender, race, contact lens wear, ocular surgery, environmental stimuli, such as temperature, humidity, air conditioning, air currents, air pollution, i.e., smoke and other atmospheric irritants, daily activities and alcohol intake [48, 49]. The most frequently used diagnostic test to determine tear film stability is tear break-up time (TBUT) after fluorescence instillation, which was first introduced by Norn, in 1969, and until now remains the gold standard in routine clinical practice [45, 48, 49]. The non-invasive break-up time (NIBUT) has also been reported because of the poor reproducibility of TBUT measurements. The NIBUT method does not involve instillation of fluorescein, blinking should be natural, not forced or suppressed, and there should be no contact between the measuring instrument and the eye or eyelids, thus objectively evaluating the observed illuminated grid pattern in an interblink interval [48]. 

### Special considerations in the pediatric population (neonates, infants, children)

Ocular anatomical features, as well as quantitative and qualitative parameters of the tears are reported to differ by age.

A rapid increase in horizontal, i.e., from canthus to canthus, and vertical, i.e., at the widest point between the eyelids, *dimensions of the palpebral aperture* has been described from birth to 1 year of age [50]. In particular, the length to width ratio was approximately 1:2 at birth, increasing to 1:2.5 by the end of the first year as measured in 64 full-term neonates and infants with a mean (SD) postnatal age of 27.5 (15) weeks (range 3.4–52 weeks) [50]. Moreover, an approximately 50% increase of the mean exposed ocular surface area was observed from the first 17 weeks of life compared with 36–53 weeks postnatally (*p* < 0.01, Kruskal–Wallis one-way ANOVA with Dunn’s post-hoc test). This presumably increases the available area for tear evaporation [50]. 

*Tear secretion* in neonates is lower than in children, as reported in a recent meta-analysis providing data from 1077 pediatric participants [51]. Overall basal tear secretion (Schirmer I test with anesthesia) was 16.3 mm per 5 min [95% Confidence Interval (CI): 13.1, 19.4] in children (1–18 years), while only 9.4 mm per 5 min (95% CI: 6.5, 12.2) in neonates (0–29 days). Similarly, overall total tear secretion (Schirmer I test without anesthesia) was 29.3 mm per 5 min (95% CI: 27.7, 30.9) in children and 17.6 mm per 5 min (95% CI: 12.0, 23.2) in neonates [51]. Although these estimates incorporate significant heterogeneity (I^2^ ≥ 98%) and should be interpreted with caution, these differences can presumably be attributed to an immaturely developed lacrimal gland during the first month of life [51, 52]. 

The *tear volume* appears to be lower during infancy compared with adulthood. A median tear volume (interquartile range) of 1.1 µL (0.40–2.38 µL), 2.5 µL (1.40–7.75 µL), and 6 µL (2.73–12.75 µL), was ascertained in 40 full-term newborns within the first 48 h of life, 14 infants within the next 4 months, and 22 adults with a mean (SD) age of 24.95 (3.63) years, respectively [53]. Of note, in 22.5% of the newborns, the tear volume was undetectable [53]. On the contrary, a negative association (Spearman correlation coefficient: −0.27, *p* < 0.01) of tear meniscus height with age was detected in 160 healthy subjects, from childhood to older adulthood, using anterior segment Fourier-domain OCT images [54]. The *tear meniscus height* of 24 healthy children, with a mean (SD) age of 10.94 (4.3) years [55], and of 248 myopic children, with a mean (SD) age of 12.26 (1.86) years [56], was 0.25 (0.16) mm and 0.22 (0.03) mm in average, as measured with slit-lamp biomicroscopy or Keratograph 5M [55, 56]. 

The *composition of tear film* also varies with age. In particular, the preocular tear lipid layer during the first week of life presented higher levels of thickness, according to the interferometry pattern, compared with that of adults, while all infants studied at 3 and 6 months postnatally had the thickest lipid layer classification of level 9 [57, 58]. It is assumed that the thicker tear lipid layer in neonates contributes to an increased tear film stability, thus preventing the thin aqueous layer from evaporation [57, 59, 60]. The increased tear film stability in neonates was also ascertained by the recent meta-analysis, where the pooled estimate for NIBUT was 21.8 Sect. (95% CI: 20.4, 23.1) in children and 32.5 Sect. (95% CI: 31.8, 33.2) in neonates [51]. 

The influence of aging on the *ocular secretory immune system* has also been recognized [61, 62]. Immunoglobulin A (IgA) tear concentration was significantly lower in full-term newborns compared with infants and adults (*p* < 0.001, Analysis of variance; *p* < 0.05, Bonferroni) [53]. However, IgA deficiency during the neonatal period may be offset by the presence of lysozyme in levels equivalent to those found in adults [50, 53, 62, 63]. The concentration of lactoferrin in full-term infants also equals to that detected in adults. Additionally, the overall mean (SD) protein concentration did not differ among newborns [10.95 (5.51) µg/µL], older infants [12.93 (3.99) µg/µL], and adults [13.04 (3.46) µg/µL] (*p* > 0.5, Analysis of variance) [53]. 

The acidic shift of the neonatal *tear pH* was ascertained in 1985, by Dahl et al., who reported a mean (SD) pH value of 6.74 (0.26) in the right and 6.87 (0.27) in the left eye of 173 newborns. This shift could be explained by the large proportion of time newborns spend sleeping and their reduced turnover rate, and is in line with adult measurements following prolonged eye closure [59, 64]. On the contrary, *tear osmolarity* was found within adult normal range (275 to 316 mOsm/L) in preterm infants measured during the first weeks of life, at term stage and at three months postnatally [mean (SD) 296.0 (19.1), 302.8 (18.0), 301.1 (20.9) mOsm/L, respectively] [65]. 

Finally, infants and children do not blink as often as adults, i.e., 12–20 blinks per minute [50, 66, 67]. The spontaneous *blinking* of full-term neonates and preschool-aged children was determined non-invasively using videographic recordings of at least 3-minute duration, as presented in Table 1 [50, 68]. Overall, a positive association of blinking rate with age was observed (Spearman correlation coefficient: 0.46, *p* < 0.01) [50, 66, 67]. These age-related variations of blinking rate may be attributed to the developmental immaturity of the neural circuits for blink control and may be associated with the thicker tear lipid layer, the increased tear film stability, and the smaller palpebral aperture in younger ages [50, 68]. 

**Table 1:** Reported blinking rates at different ages

Author, year	Population	Age	Blinking rate, mean (SE)	Interblink time, mean (SE)
Lawrenson 2005 [50]	Alert full-term neonates & infants	3.4 to 52 weeks PNA	3.6 (0.3) blinks/min	21.6 (2.8) sec [range: 6.8–82 s]
		17 weeks PNA	2 blinks/min	NR
		36–53 weeks PNA	5 blinks/min	NR
Lavezzo 2008 [68]	Newborns	30 days PNA	6.2 blinks/min	NR
	Preschool-aged children	4–6 years old	8 blinks/min	NR
*SE* standard error, *PNA* postnatal age, *NR* not reported				

### Reflexes and biochemical properties of tears in preterm compared with full-term infants

Τhe effect of prematurity on tear production constitutes a subject of investigation for more than a century [62, 69–71]. Although the results were initially controversial, more recent studies ascertain that preterm infants do secrete tears but have significantly *reduced basal and reflex tear secretion* compared with full-term infants [72–74]. Therefore, preterm infants are more vulnerable to toxicity of topically applied drugs or to corneal dryness during ophthalmological examinations, while the diagnosis of an underlying nasolacrimal duct obstruction may be masked [71, 73]. 

Preterm infants may also show *reduced levels of antimicrobial proteins* in tears, e.g., lysozyme, lactoferrin, and IgA, thus being more susceptible to infection. In particular, the concentration of tear lysozyme was found to be positively associated with both gestational age and birth weight, considering that lysozyme activity was measured to be greater in full-term infants (mean value 27.3 mm zone of lysis), compared with both preterm (mean value 24.3) and small for gestational age infants (mean value 23.4 mm) [63]. 

Finally, the Primary Jones Dye test was performed to assess the *patency* of the nasolacrimal drainage system in 24 preterm (mean gestational age: 30.5 weeks) and 25 full-term infants (mean gestational age: 39.4 weeks) at 4 weeks postnatally [74]. The test was positive in 84% of the eyes of full-term and only in 20.8% of the eyes of preterm infants (*p* < 0.001, Chi-squared test) [74]. Of note, all the eyes of the preterm infants with positive Primary Jones Dye test had corneal epithelial defects or severe superficial punctuate keratopathy [74]. Therefore, a patent nasolacrimal system in preterm infants may raise concerns regarding both the ocular surface status and the systemic absorption of topically instilled drugs.

## Pharmacokinetics of instilled drugs

The ocular surface and its adnexa compose the *anatomical and physicochemical barriers* of the eye that render the organ largely impermeable to external factors [3, 75]. Delivery of drugs via topical administration remains a significant challenge, aiming for the drug to reach the targeted tissue in sufficient amounts, while minimizing drug-induced adverse events [4]. 

Ocular bioavailability of topically instilled drugs depends on the amount of drug available onto the ocular surface and is positively associated with the *precorneal residence time* (contact time) [3, 76]. Topical instillation initiates a dynamic process consisting of reflex blinking, increased tear drainage until returning to the baseline tear volume, and induced lacrimation with subsequent drug dilution [76, 77]. These parameters, combined with enzymatic metabolism or binding of drug molecules with proteins in the tear fluid, especially the protein-rich mucin layer, as well as the drug absorption into non-targeted tissues, can negatively affect the bioavailability of topically applied drugs [77]. Optimization of the physicochemical properties of ocular drugs aim to lengthen the contact time of the drug with the precorneal tear film [75, 76]. 

Two main routes of preocular drug permeation have been described, *the corneal and the non-corneal route* [75]. The drug needs to pass the structural corneal layers, that are of variable permeability, to reach the anterior chamber. The epithelium and endothelium are both hydrophobic layers consisting of cells linked by tight junctions, restricting the passage of large molecules [75, 76]. The hydrophilic stroma consists of closely packed collagen and is the main barrier to hydrophobic agents that easily traverse the epithelium [75, 76]. Through the non-corneal route, the drug must penetrate the conjunctiva, Tenon’s tissue, and sclera to reach the anterior segment, however, less is known about the permeability of these anatomical structures [75]. Permeation through the conjunctiva seems to be enabled by the conjunctiva’s greater surface area, larger pore size, and increased hydrophilicity [75, 78, 79]. Mucus production by conjunctival goblet cells reduces drug penetration, while conjunctival lymphatics and vasculature enhance non-targeted absorption and systemic loss of drug [75, 78, 79]. 

### Preclinical studies

Preclinical studies, especially in *rabbit models*, allowed clarification of precorneal drug distribution and drug penetration characteristics through various ocular tissues [80]. 

Rabbits are an ideal experimental animal model for assessing ocular pharmacokinetics as they are easy to handle and they share common anatomical and biochemical features with humans, thus permitting extrapolation to human pharmacokinetics [80, 81]. 

Animal studies have been primarily conducted on albino rabbits, either awake or anesthetized, whose drainage ducts were either unobstructed or plugged [77, 82, 83]. Aqueous humor concentration of pilocarpine nitrate in unanesthetized rabbits with unobstructed drainage ducts peaked at about 20 min after instillation of 25 µl of 1 × 10^−2^ M solution [77, 82]. The maximum aqueous humor concentration was up to 1.33 times higher after anesthesia and duct obstruction. Considering that anesthesia is assumed to reduce or eliminate tear production in rabbits, and the profound effect of drainage duct obstruction, these results indicate the *negative impact of both lacrimation and tear drainage on ocular bioavailability* [77, 84]. In another experiment, addition of different amounts of rabbit serum albumin to the instilled pilocarpine nitrate solution, prior to administration, resulted in an even two-fold reduction of the induced miotic activity on male albino rabbits [83]. Thus, it was ascertained that the ocular bioavailability of instilled drugs can be reduced by *drug-protein binding* onto the ocular surface. This is a considerable effect, given that the high turnover rate of the tear fluid is assumed to result in constant supply of new proteins. Certain disease states, particularly inflammatory conditions, also result in increased levels of the available proteins on the ocular surface [83]. 

*Drug diffusion across the cornea*,* conjunctiva*,* and sclera* was investigated in New Zealand albino rabbits, showing that the cul-de-sac is not only a reservoir of instilled drugs [78, 79]. The non-corneal permeation route, through conjunctiva and sclera, were proved viable for ocular drug delivery, while conjunctival absorption emerged as a factor of non-productive, systemic drug loss [78, 79]. In vitro experiments, after enucleation of the entire rabbit eye, showed that b-blockers of varying lipophilicity penetrated the isolated scleral membrane more than the corneal membrane [78]. Additionally, permeation of polyethylene glycols, of varying molecular weight, showed that conjunctival permeability was less affected by the molecular size compared with corneal permeability [79]. The palpebral and bulbar conjunctivas showed equal permeabilities, approximately 15 to 25 times higher than the respective corneal permeability [79]. Moreover, the scleral permeability was approximately half of the respective conjunctival, and approximately 9 times more than the respective corneal permeability [79]. In vivo investigations, by measuring the precorneal drug clearance after punctal plugging, showed that the cornea offers substantially more resistance to polar (hydrophilic), macromolecular substances (e.g., inulin), compared to the conjunctiva and this can be attributed to the larger pore size and the higher pore density of the conjunctival epithelium [78]. 

The *pharmacokinetic profile* of a number of topically instilled drugs has also been determined on New Zealand white rabbits [85, 86]. In particular, Zhao et al. measured tropicamide and phenylephrine concentrations in rabbit ocular tissues (cornea, aqueous humor, iris/ciliary body) and plasma, after topical instillation of three doses of a 50 µl eyedrop containing phenylephrine 0.5% and tropicamide 0.5% [85]. In all cases, the maximum ocular tissues’ concentrations were much higher than plasma concentrations [85]. Additionally, Naageshwaran et al., measured the concentration of dexamethasone in rabbit tear fluid, cornea, and aqueous humor, after topical instillation of three different commercial formulations of dexamethasone [86]. Authors came to the conclusion that the aqueous humor bioavailability depended on the contact time of each formulation with the ocular surface [86]. 

### Clinical studies

Limited pharmacokinetic data of instilled drugs on humans have been reported due to difficulties in sampling intraocular tissues or fluids [2]. *Limitations* also exist regarding the need for a sufficient number of samples to adequately characterize absorption, distribution and elimination processes [2]. Sparse-sample analysis, by generating pooled pharmacokinetic parameters, is considered an applicable and acceptable way to circumvent these limitations, and becomes even more appropriate in the pediatric population. However, caution should be taken in the applied methodology to avoid compromising the precision of the estimated parameters and the validity of the results [87].

Data on *intraocular penetration* of instilled antibiotics, including chloramphenicol, netilmicin, tobramycin, and vancomycin, as well as cyclosporin A, phenylephrine 10%, tropicamide 0.5%, and metipranolol were reported after measuring the drug concentration in aqueous humor samples taken from patients undergoing cataract surgery, with or without vitrectomy [88–92]. After a single administration of suspensions containing either chloramphenicol 0.5%, or netilmicin 0.3%, or tobramicyn 0.3%, combined with corticosteroids, netilmicin and tobramycin were undetectable in the anterior chamber, whereas chloramphenicol reached a peak concentration at 120 min post-instillation and remained detectable for about 200 min [88]. Topically instilled vancomycin, in a final concentration of 30–33 mg/ml, reached therapeutic aqueous humor levels between two to four hours post-instillation [89]. The maximum intraocular concentration of cyclosporin A 2% was achieved at four hours post-instillation, though at lower levels than that found in aqueous humor after oral administration of the drug [90]. Finally, metipranolol reached maximum aqueous humor concentration at one-hour post-instillation of either 0.1% or 0.3% eyedrops [92]. 

Instilled drug overflow and spillage onto the cheek, as well as drainage of the drug through the nasolacrimal system are the major routes of instilled drug loss and *systemic drug absorption*. During the ocular drug disposition and elimination processes, several areas of drug absorption into the systemic circulation have been described, including conjunctival vessels, skin of the cheek and eyelid, nasal, oral, and gastrointestinal mucosa. Notably, the proportion of drug that is systemically absorbed through the conjunctival vessels and the nasal mucosa avoids the first-pass hepatic metabolism, thus mimicking intravenous administration [2, 39, 75, 80, 93]. Such systemic exposure could be especially harmful in both pediatric and geriatric patients considering their reduced tolerance and fragility. For instance, systemic absorption of adrenergic agonists, such as phenylephrine, has been associated with increased blood pressure, tachycardia, apnea, and gastrointestinal adverse events, including feeding intolerance, abdominal distention, paralytic ileus, even necrotizing enterocolitis. Likewise, systemic absorption of muscarinic antagonists, such as atropine or cyclopentolate, has been associated with increased blood pressure and neurological complications, including myoclonic seizures, while beta-blockers have been associated with bradycardia [94–98]. 

### Special considerations in the pediatric population (neonates, infants, children)

Anatomical and physicochemical particularities in the pediatric population seem to have an impact on both the therapeutic efficacy and the potential toxicity of instilled drugs [99, 100]. 

The narrower palpebral aperture of the very young patients results in shorter preocular residence time of the instilled drug because of *increased overflow* from the conjunctival sac and spillage onto the skin. On the contrary, the lower blink rate and the increased interblink time prolongs the drug’s contact with the ocular surface. The age-related reduction in tear volume combined with the reduced reflex tear secretion in younger patients limit the dilution of instilled drugs, resulting in *a higher initial drug concentration* and a larger quantity of drug that is drained through the nasolacrimal system, thus increasing the risk of systemic absorption and adverse events [99, 100]. However, we should not disregard that irritation from eye drop instillation is even more pronounced in the pediatric population, especially school- or pre-school-aged children, where the reaction to the procedure is also guided by emotional responses (e.g., fear) that precipitate crying, exacerbating reflex lacrimation and subsequently drug dilution and spillage onto the cheek.

Considering that instilled ocular formulations are usually pH adjusted in the range of 7.0 to 7.7 [80], the acidic shift of the neonatal tear pH, or even slight changes in tear pH, can affect the instilled drug solubility and subsequently the ocular drug penetration [93]. The latter could also be affected by the qualitative and quantitative differences of the tear protein profile in younger patients. Reduced protein levels may result in *higher free drug concentration within the tear fluid* and subsequently increased permeability, considering that drug bound to protein is unable to permeate the cornea [83, 93, 101]. 

The limited pharmacokinetic data in the pediatric population due to ethical restrictions or blood sampling considerations, complicates further decision making for rational pediatric ocular dosing to prevent drug’s toxicity [4]. One estimate is that a newborn may require half of the adult dose to obtain equal aqueous humor concentration of an instilled medication. Similarly, two thirds of the adult dose may be required at 2 to 3 years of age, and 90% of the adult dose at the age of 5 to 6 years [99]. Animal studies in newborn rabbits at different ages also ascertained an increased corneal permeability with younger age [102, 103]. However, ocular dosing in everyday practice is usually neither age- nor weight-adjusted. Subsequently, the low body mass, the immature metabolizing capacities and pharmacokinetic processes in this population result in higher plasma levels of the circulating drug, *increasing systemic exposure* and rendering them more vulnerable to the instilled drug’s toxicity [104–106]. 

### Characteristics of eyedrop formulations

Eyedrops are typically available in sterile forms of solutions, emulsions, or suspensions, that contain one or more active ingredients and various biologically active excipients with distinctive properties [107]. These forms are usually isotonic, with an optimum pH at about 7.4, equal to that of natural tears [107, 108]. 

Changes in the physicochemical properties of eyedrop formulations, e.g., pH, concentration, viscosity, lipophilicity, solubility, molecular size and shape, degree of ionization, are oriented towards enhancing corneal penetration for improving intraocular bioavailability [109]. A large variety of biologically active excipients have been used for this purpose, including, (a) *viscosity enhancers* (thickening agents, i.e., natural or synthetic polymers), such as methylcellulose or polyvinyl alcohol, that increase the contact time of the drug with the cornea, (b) *permeation enhancers* (surface-active agents), e.g., the ionic surfactant benzalkonium chloride, that alters the structure of the corneal epithelium by disrupting its plasma membrane, or non-ionic detergents for achieving a balance of drug lipophilicity and hydrophilicity, or substances that cause competitive protein binding in the tear film, (c) *cyclodextrins* that increase drug solubility on cell membranes by forming inclusion complexes with active ingredients [80, 107, 108, 110, 111]. 

However, several *excipient-related adverse events* have been reported. The increased viscosity must be balanced against the potential ocular disturbances due to blurring of vision, or foreign body sensation. Notably, according to a willingness-to pay-analysis, blurring of vision was among the adverse events for which patients are willing to pay more for an eyedrop that would avoid them [112]. Ocular discomfort and toxicity have also been attributed to commonly used preservatives, that can increase irritation, reflex blinking, and induced lacrimation, accelerating drug drainage and reducing patient compliance with therapy. Physicochemical incompatibility of the drug formulation with the tear fluid, regarding pH and osmolarity, can further increase the afore-mentioned complications [4, 11, 107]. Lastly, an unpleasant bitter taste is often reported due to eyedrops’ drainage through the nasopharynx which may also compromise patients’ compliance to therapy [112]. 

### Determinants of eyedrop size

Eyedrop instillation is performed via *single-use containers* or *multi-dose dropper bottles*, which vary considerably regarding their distinctive constructional features, such as the nozzle profile, the inner and outer aperture diameters, or the level of rigidity [113–116]. The design and characteristics of the dropper container affect the degree of adhesion of the solution to the dispenser orifice, the surface area of interaction provided by the orifice prior to release, and the rate of drop formation [115, 117–119]. These parameters, combined with the physiochemical properties of the eyedrop formulation, i.e., the surface tension, viscosity, density, temperature, and cohesion forces, as well as the number of drops previously expelled from the unit, and the administrator’s handling manners, e.g., the angle at which the dropper is held, can largely influence the instilled drop volume [113–115, 117, 120, 121]. 

The average drop volume expelled from commercially available dispensers ranges from approximately *25 µl to 70 µl*, as measured in laboratory conditions [113, 114, 122–125], while the mean repeatability coefficient across the different containers was calculated at 5.07 µl, ranging from 2.24 µl to 10.76 µl [114]. We can reasonably assume that such variations are even more evident in real-world clinical practice, where administrators’ manipulations are less predictable. Patient-survey data ascertain the unnecessarily large size of the instilled drops and the redundant dispensation of more than one drop at a time, which contribute to early exhaustion of the eyedrop bottle, even in individuals familiar with self-administering eyedrops, e.g., glaucoma patients [126–128]. This *exhaustion and wastage* result in inadequate amount of medication available between prescription refills and can negatively affect patient compliance with therapy [126, 127]. 

## Rationale for using microdrops

**Fig. 1:**
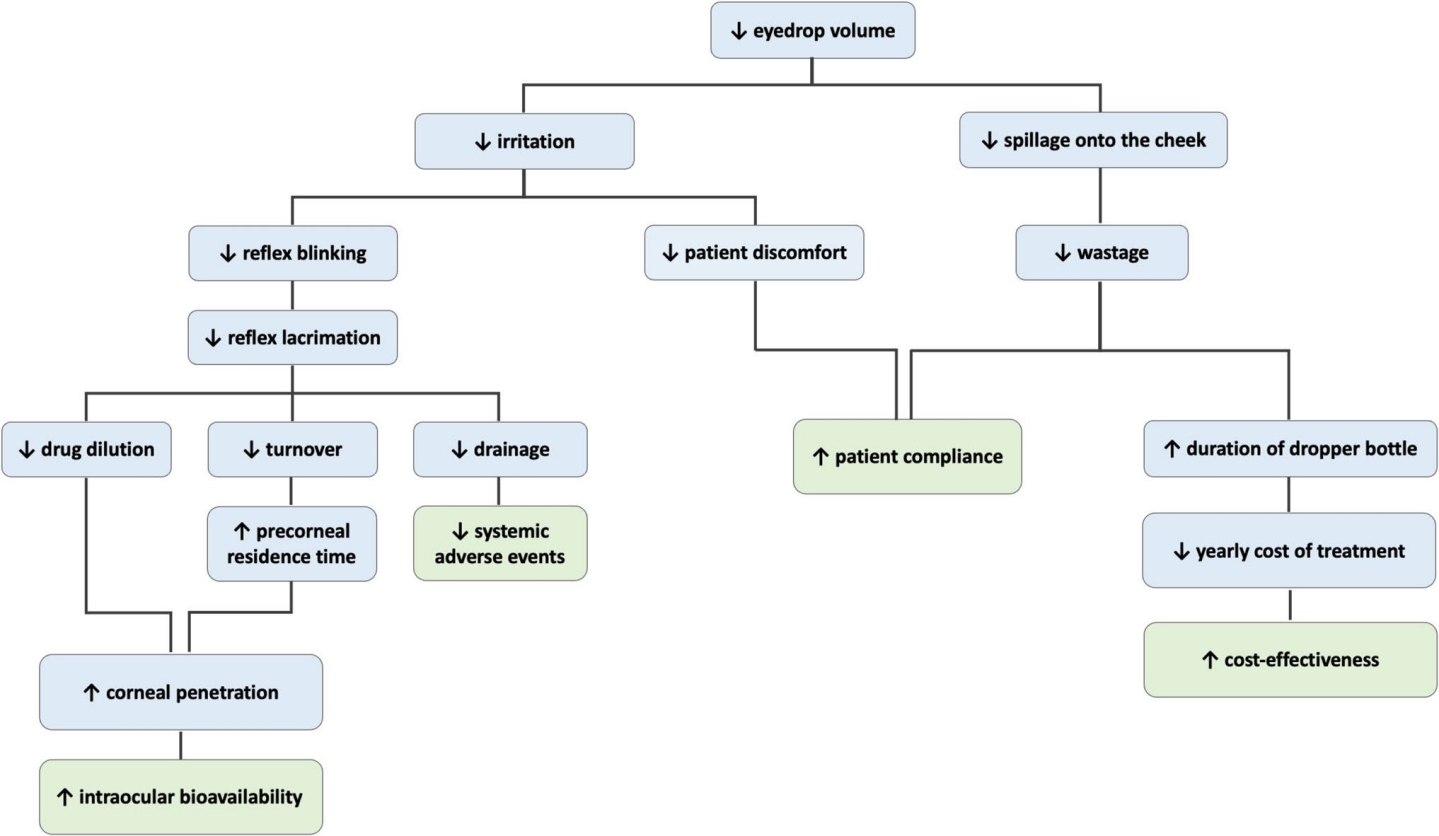
Schematic diagram depicting the rationale for using microdrops

The reduction of the instilled drop volume, i.e., the use of microdrops, emerged in the literature already in the ‘70s. It was brought forward as a promising method of enhancing corneal penetration and improving intraocular bioavailability of topically applied drug formulations, while minimizing excipient-related adverse events and systemic toxicity. The rationale for using microdrops is illustrated in the schematic diagram of Fig. 1 and is primarily based on the following parameters: a) reflex blinking after instillation increases drainage and spillage onto the cheek in a volume-dependent manner, leading to less available drug to exert a local effect or to be absorbed into the eye, b) although the maximum quantity of fluid that could be contained in the cul-de-sac, in the upright position, without overflow, is about 30 µl, if blinking is allowed the eye can only hold approximately 10 µl, c) on the contrary, an extremely small instilled drop volume is greatly diluted by tears and is washed out more quickly [10, 12, 129]. 

### Animal studies investigating reduced eyedrop volume

Investigation of lacrimal and instilled fluid dynamics in rabbit eyes has proved that the *rate of drug loss*, via both drainage and spillage onto the cheek, is *volume-dependent* and increases linearly with the instilled drop volume [10]. The fraction of the applied dose absorbed into the rabbit eye has been shown to be greater as the volume of the instilled drug solution is decreased, and, similarly, the miotic activity of pilocarpine was enhanced by administering the same amount of drug in reduced drop volume [10, 17]. 

### Clinical applications of microdrops

Brown and Hanna in 1978 were the first to attempt applying microdrops in clinical practice [11]. They assessed the effect of 5 µl and 10 µl of combined phenylephrine 5% and tropicamide 0.5%, compared with standard drops (70 µl) in adults. The results were promising, showing no difference in mydriasis after microdrop instillation, while tearing and ocular irritation were experienced only after standard drop instillation [11]. Several studies have been conducted thereafter, investigating a variety of ocular drugs including mydriatics, cycloplegics, and ocular hypotensive drugs, as detailed below and in Tables 2 and 3.

**Table 2:** Summary of controlled trials evaluating the use of reduced eyedrop volume compared with standard drops

Author, year	Design	Population (sample size)	Regimen(s)	Instilled drop volumes (level of comparison, i.e., eyes or subjects)	Method of instilling microdrops	Efficacy outcome(s)	Safety outcome(s)
a1-adrenergic agonists and muscarinic antagonists							
Brown 1978 [11]	Non-randomized controlled trial	Adults (41)	Group A - µ-drop of **PHE 5% & TRO 0.5%**, in a mixture, in one eye (1 drop, 3 doses, 5 min interval) - s-drop of **PHE 10% & TRO 1%**, sequentially, in the other eye (1 drop, 3 doses, 5 min interval) Group B - µ-drop of **PHE 5% & TRO 0.5%**, in a mixture, in one eye (1 drop, 3 doses, 5 min interval) - s-drop of **PHE 10% & TRO 1%** in the other eye (1 drop, 3 doses, 5 min interval)	A) 5 µl vs. 70 µl (in either eye of the same subject) B) 10 µl vs. 70 µl (in either eye of the same subject)	A) Sterile PE-160 polyethylene tubing wrapped at least four times around the barrel of a 1-ml tuberculin plastic syringe and attached to the syringe through an 18-gauge needle B) Calibrated micropipette	A) Mean pupil diameter at T45 increased from 2.09 mm to 7.10 mm after µ-drop, and from 2.10 mm to 7.39 mm after s-drop B) No difference in mydriasis between µ-drop and s-drop	Tearing and ocular irritation experienced only after s-drop (percentages are not reported)
Brown 1987 [130]	Crossover RCT	Adults (10)	- µ-drop of **PHE 10% ** (1 drop, 2 doses, 10 min interval) - s-drop of **PHE 2.5%** (1 drop, 2 doses, 10 min interval)	8 µl vs. 32 µl (subjects, at least one-week washout period)	Calibrated micropipette	Larger increase from baseline in pupil diameter at T45 after µ-drop (*p* = 0.0001)	No difference in the average plasma concentration of PHE at T10, T20, T40
Lynch 1987 [13]	RCT	Preterm infants (11 for efficacy and 17 for safety outcome)	**PHE 2.5%** (1 drop, 3 doses, 2–5 min interval)	8 µl vs. 30 µl (eyes for the efficacy and subjects for the safety outcome)	Calibrated micropipette	No difference in the percent change from baseline in pupil diameter at T60	Lower plasma levels of PHE at T10 after µ-drop (*p* = 0.01)
Craig 1991 [131]	Crossover non-randomized controlled trial	Healthy volunteers (20)	**PHE 10%** (regimen NR)	10 ± 2 µl vs. 30 ± 4 µl (subjects, one week washout period)	22G Venflon iv cannula attached to the end of a Minim after removal of the central stylette	No difference in the change from baseline in pupil diameter at T60	Less discomfort was self-reported after µ-drop (percentages are not reported)
Gray 1991 [132]	RCT	Patients requiring diagnostic pupil dilation (60)	Group A: **PHE 10% & TRO 1%** (1 drop of each, 2 min apart) Group B: **TRO 1%** (1 drop) Group C: **TRO 0.5%** (1 drop)	5 µl vs. 26 µl (in either eye of each subject in all three groups)	30G Rycroft cannula attached to the minim	A) Larger increase in pupil diameter at T20 after s-drop B) No difference in the change from baseline in pupil diameter at T20 C) Larger increase in pupil diameter at T20 after s-drop	Less ocular discomfort was reported after µ-drop (percentages are not reported)
Gray 1992 [133]	Crossover non-randomized controlled trial	Healthy adult volunteers (20)	**TRO 1%** (Regimen NR)	5 µl vs. 26 µl (subjects; only one eye was tested in each individual, at least one-week washout period)	Narrow bore cannula attached to the minim	No difference in the change in pupil: cornea diameter ratio at T30, T60, T120 and T240	Improved rate of recovery of distance and near visual acuity at T30 after µ-drops compared with s-drops (*p* < 0.05)
Noske 1993 [14]	Crossover non-randomized controlled trial	Children with uncorrected or undercorrected hypermetropia (15)	**Atropine 0.5%** (< 2 years) − 1 µ-drop, 3 doses, 5 min interval − 1 s-drop, 6 doses (twice daily for 3 days) **Atropine 1%** (> 2 years) − 1 µ-drop, 3 doses, 5 min interval − 1 s-drop, 6 doses (twice daily for 3 days)	5 µl vs. 30–36 µl (eyes, without washout period)	Calibrated micropipette	Higher by 0.25 D mean spherical equivalent of cycloplegic refraction at T90 after s-drop (*p* < 0.05) No difference in cylindrical power and axis after s-drop and µ-drop	Increased change from baseline in HR at T90 after s-drop (*p* < 0.05) Typical flushing observed in some children after both s-drop and µ-drop
Wheatcroft 1993 [134]	Non-randomized controlled trial	Preterm infants (26)	**PHE 2.5% & CYCLO 0.5% ** (1 drop of each, 5 min apart)	5 µl vs. 26 µl (in either eye of the same subject)	30-gauge Rycroft intraocular cannula attached to the minim	No difference in pupil diameter at T40-T60	Not assessed
Whitson 1993 [12]	Crossover RCT	Healthy adults (13)	**PHE 10%** (1 drop, 2 doses, 10 min interval) +/- eyelid closure for 1 min	10 µl vs. 30 µl (subjects, at least one-week washout period)	Calibrated micropipette	No difference in pupil diameter at T60 For both s-drop and µ-drop, eyelid closure produced greater pupil dilation	No difference in plasma concentration at T20
Elibol 1997 [135]	Crossover non-randomized controlled trial	Infants (53)	Group A: **PHE 10% ** (1 drop, 2 doses, 5 min interval) Group B: **CYCLO 1%** (1 drop, 2 doses, 5 min interval) Group C: **TRO 0.5%** (1 drop, 2 doses, 5 min interval)	A) 5.1 µl vs. 33 µl (subjects, 3–7 days washout period) B) 6.5 µl vs. 39.2 µl (subjects, 3–7 days washout period) C) 5.4 µl vs. 33.8 µl (subjects, 3–7 days washout period)	24-gauge intravenous cannula attached to the end of a minim after removal of the central stylette	A) No difference in pupil diameter at T15, T30, T45, T60 B) No difference in pupil diameter at T15, T30, T45, T60 C) Increased pupil diameter at T30, T45, T60 after s-drops compared with µ-drops (*p* < 0.01)	A, B, C) Increased change from baseline in mean BP at T15, T30, T45, T60 after s-drop (*p* < 0.01) A, B, C) No difference in the change from baseline in HR at T60 B, C) Significant flushing after s-drop of CYCLO and TRO compared with µ-drop (*p* < 0.01)
Ianchulev 2016 [136]	RCT	Healthy adults (102)	**PHE 2.5% & TRO 1%**, sequentially Group A: 1 µ-drop * 1.5 µl of each drug in one eye Group B: 2 µ-drops * 3 µl of each drug in one eye (3-min interval) Group C: 1 µ-drop * 6 µl of each drug in one eye In all three groups: 1 s-drop (26 µl) of each drug in the other eye	1.5–3 µl or 6 µl vs. 26 µl (in either eye of the same subject)	Piezoelectric device	No difference in pupil diameter at T10, T20, T60 min after instillation of 2*3 µl and 1*6 µl compared with s-drops	Participants preferred piezoelectric self-delivery to s-drops, reporting better head-positioning comfort, reduced tearing/overflow and increased likelihood of adhering to ocular medication regimens (*p* < 0.0001)
Ianchulev 2018 [137]	Crossover non-randomized controlled trial	Healthy adults (12)	Group A: s-drop of **PHE 10%** (Regimen NR) Group B: s-drop of **PHE 2.5%** (Regimen NR) Group C: µ-drop of **PHE 10%** (Regimen NR)	8 µl vs. 32 µl (subjects, one-week washout period)	Microdroplet spray device	No difference in the change from baseline in pupil diameter at T30, T45, T60 µ-drop of PHE 10% achieved comparable pupil dilation as s-drop of PHE 10%, and superior pupil dilation to s-drop of PHE 2.5% at T75 (*p* = 0.009)	No difference in HR and BP at T60 No difference in PHE plasma levels at T20 after µ-drop of PHE 10% or s-drop of PHE 10% Higher PHE plasma levels at T20 after µ-drop of PHE 10% compared with s-drop of PHE 2.5% (*p* = 0.021)
Seliniotaki 2022 [125]	Pilot crossover RCT	Preterm infants (25)	**PHE 1.67% & TRO 0.33%** (mixture) (1 drop, 3 doses, 5 min interval)	6–7 µl vs. 28–34 µl (subjects, one-week washout period)	27-gauge irrigating cannula attached to a disposable 2.5 ml syringe	No difference in pupil diameter at T45, T90, T120	No difference in HR at T45, T90 Lower levels of HR at T120 after µ-drop (*p* = 0.046) No difference in BP and oxygen saturation at T45, T90, T120 No difference in 24-h cardiorespiratory and gastrointestinal AE
Hoppe 2023 [138]	Non-inferiority RCT	Children (50)	**PHE 2.5% & CYCLO 1% & TRO 1%** (1 drop of each drug sequentially)	10.4 µl vs. 50 µl (eyes)	Nanodropper adaptor	Inconclusive trial regarding spherical equivalent and pupil constriction percentage^**^ at T30 Non-inferiority of µ-drop compared with s-drop regarding pupil diameter at T30 (non-inferiority margin: −0.20)	Inconclusive trial regarding IOP after dilation
Seliniotaki 2024 [139]	Non-inferiority crossover RCT	Preterm infants (83)	**PHE 1.67% & TRO 0.33%** (mixture) (1 drop, 3 doses, 5 min interval)	6.5 µl vs. 28–34 µl (subjects, at least one-week washout period)	Calibrated micropipette	Superiority of µ-drop regarding pupil dilation at T45 (*p* = 0.008) Non-inferiority of µ-drop regarding pupil dilation at T90 and T120 (Bonferroni-corrected 95%CI at T90: −0.10, 0.17; at T120: −0.18, 0.14; non-inferiority margin: −0.4 mm)	Lower levels of oxygen saturation at T45 (*p* = 0.03) and T90 (*p* = 0.04) after s-drop Higher percentage of 24 h hypertensive episodes (*p* = 0.01) after s-drop Pooled pharmacokinetic data of PHE within 3-hours post-instillation, insufficient to compare µ-drop with s-drop
beta-blockers							
Montoro 1990 [94]	RCT	Adults (20)	**Timolol maleate 0.5%** (Regimen NR)	29.12 ± 1.2 µl vs. 49.26 ± 1.4 µl (subjects; before-after)	NR	Significant reduction in IOP in each separate group (time-point not specified)	Decrease in HR from baseline in each separate group – larger decrease observed after s-drop (0.05 < *p* < 0.10) No difference in BP compared with baseline in each separate group
Steger 2024 [98]	Non-inferiority RCT	Adults with open angle glaucoma and ocular hypertension (419)	**Timolol maleate 0.5%** (1 drop)	12.5 µl vs. 28 µl (subjects)	Nanodropper adaptor	Significant reduction in IOP in each separate group at all time-points (1-, 2-, 5- and 8-hours post-instillation) Non-inferiority of µ-drop regarding IOP at 1-, 2-, and 8-hours post-instillation (non-inferiority margin: 1.5 mmHg) Inconclusive trial at 5-hours post-instillation	Significant reduction in systolic BP in each separate group at 1-, 2-, and 5- hours post-instillation) No difference in diastolic BP change from baseline in each group at any time-point Greater absolute and relative HR decrease from baseline after s-drop
a2-adrenergic agonists							
Petursson 1984 [15]	Crossover RCT	Adults (16)	Placebo in one eye & **Clonidine 0.25%** or **Clonidine 0.5%** in the other eye (1 drop)	15 µl vs. 70 µl (eyes)	Calibrated pipette	Both s-drop and µ-drop of 0.25% and 0.50% clonidine lowered the IOP at 1–5 h compared with the placebo	Only s-drop of 0.50% clonidine lowered systemic BP at 1–5 h Transient “burning in the eye” after µ-drop of clonidine 0.25% (one patient)
Vocci 1992 [140]	Crossover RCT	Healthy adult volunteers (29)	**Apraclonidine 0.5% ** (1 drop)	16 µl vs. 30 µl (subjects, one-week washout period)	A potentially commercially available eyedrop bottle that delivers 16 µl	No difference in mean IOP at 1, 3, 8, 12 h Reduced IOP at 1, 3, 8, 12 h after s-drop and µ-drop compared with placebo	No difference in the % change from baseline in BP No difference in the number of subjects experiencing AE including blurred vision, dry mouth, drowsiness, tiredness
muscarinic agonists							
File 1980 [16]	Crossover RCT	Healthy adult volunteers (10)	**Pilocarpine 0.5%** (1 drop)	20 µl vs. 50 µl (subjects; only one eye was tested in each individual, one-week washout period)	Micropipette with sterile disposable tips	No difference in the change from baseline in pupil diameter up to 2 h post-instillation	Not formally assessed
Lal 1995 [141]	Crossover RCT	Healthy volunteers (12)	**Pilocarpine 2%** (1 drop) after the drop administration, the inner punctum was pressed for 1 min	10 µl vs. 20 µl vs. 40 µl vs. 80 µl (subjects, 7-days washout period)	Micropipette	Larger decrease in pupil diameter at T15, T30, T45, T60, T90, T120, T180, T240, T300 after 10 µl, compared with 20 µl, 40 µl and 80 µl	No difference in HR at T15, T30, T45, T60, T90, T120, T180, T240, T300 Fewer subjects experiencing frontal headache or ocular irritation after 10–20 µl (statistical analysis not performed)
allogeneic serum							
Vermeulen 2023 [142]	Multi-center non-inferiority crossover RCT	Adults with dry eye disease (49)	**Allogeneic serum** (6 drops daily for 1 month)	7 µl vs. 50 µl (hospitals, one-month washout period)	mu-Drop system	Non-inferiority of µ-drop compared with s-drop regarding the Ocular Surface Disease Index score (mean difference − 2.60; 95%CI −9.16 to 3.97; *p* = 0.006; non-inferiority margin = 6)	Not formally assessed

*µ-drop* microdrop instillation, *s-drop* standard drop instillation, *PHE *phenylephrine, *TRO *tropicamide, *CYCLO *cyclopentolate, *RCT *randomized controlled trial, *BP *blood pressure, *HR *heart rate, *NR *not reported, *IOP *intraocular pressure, *AE *adverse events, *ROP *retinopathy of prematurity, *CI *confidence interval

**Pupillary constriction percentage was calculated by the pupillometer in response to a 180-mW flash: (maximum - minimum) / maximum

**Table 3:** Summary of uncontrolled trials evaluating the use of reduced eyedrop volume

Author, year	Design	Population (sample size)	Regimen(s)	Instilled drop volumes (level of comparison, i.e., eyes or subjects)	Method of instilling microdrops	Efficacy outcome(s)	Safety outcome(s)
a1-adrenergic agonists and muscarinic antagonists							
Kremer 2021 [143]	Pilot randomized uncontrolled trial	Preterm infants (16)	Group A: **PHE 1% & CYCLO 0.2%** in a mixture (1 drop) Group B: **PHE 0.5% & CYCLO 0.1%** in a mixture (1 drop)	Only microdrops – exact volume ΝR (subjects)	24-gauge cannula attached to a 3 mL syringe after removal of the needle	No difference in pupil diameter at T45 and T90	No difference in the level of respiratory support up to day 2 No difference in gastrointestinal AE
Kremer 2023 [144]	Multi-center, non-inferiority, uncontrolled, randomized trial	Preterm infants (150)	Group A: **PHE 1% & CYCLO 0.2%** in a mixture (1 drop, up to 3 doses, 20 min interval) Group B: **PHE 0.5% & CYCLO 0.1%** in a mixture (1 drop, up to 3 doses, 20 min interval)	7 µl (subjects)	24-gauge cannula attached to the end of a syringe after removal of the needle	Successful ROP eye examination in both groups Smaller pupil dilation occurred in group B compared with group A (*p* = 0.01)	No difference in cardiovascular, respiratory (up to day 2), and gastrointestinal (up to day 7) AE
beta-blockers							
Filippi 2017 [145]	Multicenter, open-label, pilot uncontrolled trial	Infants with stage 2 ROP in zone II without plus disease (23)	**Propranolol 0.1%** (3 drops every 8 h in each eye, as soon as stage 1 ROP was diagnosed and until complete retinal vascularization, but for no longer than 90 days)	6 µl	Variable volume pipette	1/23 progressed to ROP stage 2 with plus 5/23 progressed to ROP stage 3 with plus	None of the severe AE usually related to propranolol was observed No extended hospital stays Plasma propranolol during days 1–3 and on day 10 was about 10 times lower than that reported after oral propranolol administration of 1 mg/kg/d
Filippi 2019 [146]	Multi-center, open-label, single arm, phase IIB clinical trial	Preterm infants with GA ≤ 32 weeks and birthweight ≤ 1500 g, diagnosed with stage 1 ROP in zone II or III (98)	**Propranolol 0.2%** (3 drops every 6 h in each eye, as soon as stage 1 ROP was diagnosed and until complete retinal vascularization, but for no longer than 90 days)	6 µl	Micropipette	12/98 progressed to ROP stage 2 or 3 with plus Reduced number of treatments with laser or anti-VEGF compared with historical controls (*p* = 0.107)	None of the severe AEs usually related to propranolol was observed No extended hospital stays Large inter-individual differences in plasma concentrations were observed between the patients
Scaramuzzo 2023 [147]	Single arm, phase II clinical trial	Preterm infants (25)	**Propranolol 0.2%** (3 drops every 6 h in each eye, as soon as stage 1 or stage 2 ROP was diagnosed and until complete retinal vascularisation had been achieved, but for no longer than 90 days)	6 µl	Micropipette	A fourfold reduction in the number of infants who reached stage 3 ROP compared with historical controls (*p* = 0.013)	No reported AEs
prostaglandin analogues							
Pasquale 2018 [148]	Uncontrolled trial	Adults (30)	**Latanoprost 0.005%** (1 drop every morning for 2 days)	8 µl	High-precision, piezo-print horizontal delivery system	Reduced levels of diurnal IOP at 1st and 2nd day post-instillation (*p* < 0.0001)	No cases of unintentional overdosing, tear fluid overflow, or dispenser tip/nozzle touching the eye None reported ocular discomfort No reported AEs

*BP *blood pressure, *HR *heart rate, *NR *not reported, *IOP *intraocular pressure, *ROP *retinopathy of prematurity, *PHE *phenylephrine, *CYCLO *cyclopentolate, *AE* adverse event

#### Mydriatics and cycloplegics

Overall, seventeen studies investigated the efficacy and safety of microdrop instillation of a1-adrenergic agonists and muscarinic antagonists. Eight of them (8/17) were randomized controlled trials (RCTs), either full-scale (7/8) or pilot (1/8), with either parallel (4/8) or crossover (4/8) design [12, 13, 125, 130, 132, 136, 138, 139]. Two studies (2/17), a pilot (1/2) and a full-scale (1/2), were crossover randomized uncontrolled trials, without including a control group receiving the standard of care, i.e., standard drops [143, 144]. Seven studies (7/17) were non-randomized controlled trials, with either parallel (2/7) or crossover (5/7) design [11, 14, 131, 133–135, 137]. 

Seven studies were conducted in *preterm infants* requiring screening for retinopathy of prematurity [13, 125, 134, 135, 139, 143, 144], aiming to offer an alternative mydriasis technique that would minimize systemic adverse events in this fragile population [96]. Microdrops of (a) phenylephrine 2.5%, or (b) phenylephrine 10%, or (c) cyclopentolate 1%, or (d) combined phenylephrine 2.5% and cyclopentolate 0.5% instilled sequentially, or (e) combined phenylephrine 1.67% and tropicamide 0.33% in a mixture, resulted in efficient mydriasis, compared with their respective standard drops (range of compared drop volumes: 5–8 µl vs. 26–39 µl) [13, 125, 134, 135, 139]. Only tropicamide 0.5% alone was proved inferior in pupil dilation when being administered in microdrops [135]. In a recently published double-masked non-inferiority crossover RCT, microdrops of combined phenylephrine 1.67% and tropicamide 0.33% in a mixture were proven superior regarding mydriatic efficacy at 45 min post-instillation, and non-inferior at 90 and 120 min post-instillation, compared with standard drops of the same regimen administered in preterm infants undergoing ROP screening [139]. 

Regarding safety outcomes in *preterm infants*, the reported lower plasma levels of phenylephrine, at 10 min post-instillation, after microdrops [13], the decreased change from baseline in blood pressure (BP) measurements within one hour after microdrop instillation [135], the lower levels of oxygen saturation at 45 and 90 min post-instillation after standard drops [139], and the higher percentage of 24 h hypertensive episodes that were observed after standard drops [139], provide evidence for a superior safety profile of microdrops.

Two studies were conducted in *pre-school- and school-aged children*, assessing the cycloplegic effect of microdrop compared with standard drop instillation of either (a) atropine 0.5%, or (b) atropine 1%, or (c) combined phenylephrine 2.5%, cyclopentolate 1%, and tropicamide 1%, administered sequentially [14, 138]. However, the variability in the applied methodology, including the target age group, the regimens used, and the time point of assessment, preclude drawing clinically applicable conclusions.

Finally, eight studies were conducted in *adults* and, in most cases, no difference in pupil diameter after microdrop or standard drop instillation was observed [11, 12, 130–133, 136, 137]. Interestingly, near vision recovery after tropicamide 1% was reported to be quicker after microdrop instillation (*p* < 0.001) [133]. When ocular discomfort was assessed, it was found to be reduced after microdrop instillation, irrespective of the regimen used [11, 131, 132, 136]. Phenylephrine plasma concentration at either 10 or 20 or 40 min post-instillation did not differ after either standard drops or microdrops [12, 130, 137]. 

#### Ocular hypotensive drugs

A parallel RCT evaluated the effect of administering a reduced drop volume of *timolol maleate 0.5% in adults* [94]. Although the studied drop volume was in fact rather large and does not qualify as a microdrop, a larger decrease in HR was found after instilling 49.26 µl (group A) compared with 29.12 µl (group B), while the intraocular pressure lowering effect was maintained (mean decrease in IOP was 4.4 mmHg in group A and 5.3 mmHg in group B) [94]. A recently published non-inferiority, parallel RCT in 419 adults with open-angle glaucoma and ocular hypertension, showed a significant reduction in IOP after instilling either 12.5–28 µl of *timolol maleate 0.5%* at 1-, 2-, 5- and 8-hours post-instillation. The study also established non-inferiority of microdrops (12.5 µl) regarding the IOP at 1-, 2-, and 8-hours post-instillation [98]. A significant reduction in systolic BP was observed in each separate group at 1-, 2-, and 5- hours post-instillation, while there was no difference regarding the diastolic BP change from baseline in either group at any time-point. A greater absolute and relative HR decrease from baseline was reported after standard drops [98]. 

Two crossover RCTs have also been conducted in the field of glaucoma medications, ascertaining comparable efficacy for microdrops within 12 h post-instillation [15, 140]. Vocci et al. reported no difference in the percent change from baseline in systolic and diastolic BP, as well as in the number of subjects experiencing adverse events after a 16 µl drop and a 30 µl drop of *apraclonidine 0.5%* [140]. On the contrary, Petursson et al. observed a substantial decrease in BP only after standard drop instillation of *clonidine hydrochloride 0.5%* [15]. 

Several ongoing RCTs on the use of microdrops in glaucoma patients are registered in the ClinicalTrials.gov database [149–151]. Their results are expected to be even more informative considering that they will provide evidence on efficacy and safety after 6-months of treatment [149–151].

Finally, in their study, Karki et al. highlighted another advantage of using microdrops [129]. They showed that the reduction of the instilled drop volume could greatly diminish the yearly cost of treatment and lower the medication wastage of timolol maleate for glaucoma patients [129]. 

#### Other ocular drugs

Two crossover RCTs assessed the miotic activity of *pilocarpine 0.5% and 2% in healthy volunteers* [16, 141]. It appears that microdrops of pilocarpine are equally effective with a decreased number of self-reporting adverse events.

Instilled microdrops of *propranolol 0.1% and 0.2%* have been suggested as an alternative to oral propranolol *in reducing ROP progression* [145, 146]. Although propranolol 0.1% was not proved effective in the initial pilot trial, the multi-center, open-label, single arm, phase IIB clinical trial that followed ascertained that microdrops of propranolol 0.2% reduced the rate of ROP progression from stage 1 to stage 2 plus or 3 plus by 11.3%, i.e., from 23.7% in historical controls to 12.4% [145, 146]. These efficacy results were also confirmed by another research team, showing a fourfold reduction in the number of treated infants who reached stage 3 ROP compared with historical controls [147]. In the study by Filippi et al., none of the participants in the study group experienced any severe adverse event, e.g., bradycardia, bronchospasm, and severe hypotension, or any rebound effect after treatment discontinuation. Unfortunately, no information regarding safety outcomes is provided for the historical controls, and authors do not explore the role of potential confounders [146]. 

Finally, allogeneic serum eye drops in either microdrop (7 µl) or standard drop (50 µl) size were investigated in adults with dry eye disease, in a recently published multi-center, non-inferiority, crossover RCT. The study proved non-inferiority of microdrops compared with standard drops regarding the Ocular Surface Disease Index score after 1 month use [142]. 

## Conclusion

In conclusion, microdrops have been used effectively in infants, children, and adults for diagnostic and therapeutic purposes, with evidence of reduced systemic toxicity. The results should be interpreted with caution given the limitations that some of the included studies, present, e.g., inadequate sample size, existence of potential confounders, flaws in the randomization process or masking, that may introduce bias. However, risk of bias assessment was beyond the purpose of this scoping review, that was to introduce the reader to the concept of microdrops, summarize the existing data, and reveal the necessity for more research in this very promising field.

Several improvised methods of instilling microdrops have been described, and only recently a disposable device that allows microdrop instillation has become commercially available and successfully used in clinical trials. Considering the special characteristics of the marketed eyedrop formulations, we can reasonably assume that the lowest effective dose is regimen-dependent and, as such, should be studied separately for each medication. Future studies with an appropriate follow-up combined with pharmacokinetic analyses are needed for drawing more informative conclusions and for bringing microdrop instillation into everyday clinical practice.

## Appendix

Search string on PubMed

("microdrop"[All Fields] OR "microdrops"[All Fields]) OR "micro-dose"[All Fields] OR "micro-doses"[All Fields] OR ("microdose"[All Fields] OR "microdoses"[All Fields] OR "microdosing"[All Fields]) OR ("microdose"[All Fields] OR "microdoses"[All Fields] OR"microdosing"[All Fields]) OR "minidrop"[All Fields] OR ("minidrops"[All Fields] OR ("nanodrop"[All Fields] OR "nanodrops"[All Fields]) OR ("nanodrop"[All Fields] OR "nanodrops"[All Fields]) OR "nanodropper"[All Fields] OR ("reduc*"[All Fields] AND "drop"[All Fields] AND ("volum"[All Fields] OR "volume"[All Fields] OR "volumes"[All Fields] OR "voluming"[All Fields])) OR "drop size"[All Fields]) AND ("ophthalmologie"[All Fields] OR "ophthalmology"[MeSH Terms] OR "ophthalmology"[All Fields] OR "ophthalmology s"[All Fields] OR ("eye"[MeSH Terms] OR "eye"[All Fields]) OR ("ophthalmic solutions"[Pharmacological Action] OR "ophthalmic solutions"[MeSH Terms] OR ("ophthalmic"[All Fields] AND "solutions"[All Fields]) OR "ophthalmic solutions"[All Fields] OR "eyedrop"[All Fields] OR "eyedrops"[All Fields]) OR ("ophthalmic solutions"[Pharmacological Action] OR "ophthalmic solutions"[MeSH Terms] OR ("ophthalmic"[All Fields] AND "solutions"[All Fields]) OR "ophthalmic solutions"[All Fields] OR "eyedrop"[All Fields] OR "eyedrops"[All Fields]))

159 results until 29th December 2024
